# A longitudinal study on latent TB infection screening and its association with TB incidence in HIV patients

**DOI:** 10.1038/s41598-019-46570-5

**Published:** 2019-07-12

**Authors:** Ngai Sze Wong, Chi Chiu Leung, Kenny Chi Wai Chan, Wai Kit Chan, Ada Wai Chi Lin, Shui Shan Lee

**Affiliations:** 1 Stanley Ho Centre for Emerging Infectious Diseases, The Chinese University of Hong Kong, Shatin, Hong Kong China; 2 Hong Kong Tuberculosis, Chest and Heart Diseases Association, Hong Kong, China; 3 Special Preventive Programme, Centre for Health Protection, Department of Health, Hong Kong Special Administrative Region Government, Hong Kong, China

**Keywords:** HIV infections, Tuberculosis

## Abstract

Latent TB infection (LTBI) in HIV patients, its treatment, and immunological recovery following highly active antiretroviral therapy (HAART) could interact and impact TB disease progression. We aim to examine the factors associated with LTBI and TB disease development among HIV patients. Longitudinal clinical and laboratory data were accessed from the largest HIV specialist clinic in Hong Kong, where HAART and yearly LTBI screening are routinely provided for HIV patients. Between 2002 and mid-2017, among 2079 HIV patients with 14119 person-years (PY) of follow-up, 32% of LTBI screened patients (n = 1740) were tested positive. The overall TB incidence was 1.26/100 PY from HIV diagnosis to HAART initiation, falling to 0.37/100 PY. A lower risk of TB disease progression was associated with local residence, Chinese ethnicity, negative baseline LTBI result, being on HAART, LTBI treatment, higher baseline CD4 and CD4/CD8 ratio. A positive test at baseline, but not subsequent testing results, was significantly associated with TB disease development. Baseline LTBI screening is an important strategy for identifying HIV patients at risk of TB disease progression. Routine repeat LTBI screening on an annual basis might not give additional benefits to patients on HAART with good immunological responses. Such practice should require re-evaluation.

## Introduction

As of 2014, a quarter of the global population were harbouring latent tuberculosis infection (LTBI)^1^. Because of the underlying immune deficiency, HIV-infected individuals with LTBI are at 26-fold higher risk of TB reactivation^2^. Active TB disease may present as an acquired immune deficiency syndrome (AIDS) defining illness (ADI)^3^, or a co-morbid condition^2^. TB has remained the leading cause of mortality (33%) among HIV-infected individuals, though substantial reduction was observed from 2005 to 2015^4^. LTBI screening followed by treatment in HIV-infected individuals is generally recommended in guidelines in low TB burden areas such as the United States (US) and the United Kingdom (UK)^5–7^. However, screening before LTBI treatment is generally not applied in most high TB burden areas except South Africa^8^.

Practically, it is a challenge to effectively implement LTBI screening and treatment. Conventionally LTBI screening is performed by tuberculin skin testing (TST), an observer-dependent procedure requiring a second visit within 48 to 72 hours^9^. Alternatively, the more expensive one-off Interferon-γ release assay (IGRA) could be performed, which however give a non-comparable result^10^. The compliance of HIV services in implementing LTBI screening and treatment was often unsatisfactory, as noted in high-income countries with a low TB/HIV burden^11–15^. In UK, the full compliance rate of healthcare providers was just 35%^11^, while the ever LTBI testing rates among HIV patients in US, Canada and New Zealand were below 70%^12–15^. With highly active antiretroviral therapy (HAART), immune recovery is becoming an achievable outcome^16,17^. HAART could potentially slow LTBI progression, thus reducing TB disease incidence^14,18,19^. In a Canadian study, the TB incidence fell following HAART coverage expansion^14^. The need for regular LTBI screening may not be as important with HAART.

We conducted a retrospective longitudinal study in HIV patients in Hong Kong, a city with intermediate TB burden (60.5/100000 persons)^20^, low (0.9%) HIV prevalence among TB patients (https://www.aids.gov.hk/english/surveillance/sur_report/hiv16.pdf) and low TB incidence (0.59 per 100 person-years (PY)^9^) among HIV patients. In practice, yearly LTBI screening followed by treatment is part of standard recommendations for HIV patients^9^. We undertook to determine the TB incidence among the HIV positive population, examine factors associated with LTBI and TB diseases, and explore the association with baseline and subsequent LTBI testing results, and identify the predictors, including HAART, LTBI screening and treatment, of TB incidence.

## Methods

### Study subjects, settings and selection criteria

HIV-infected patients attending the Integrated Treatment Centre (ITC), the largest HIV clinical service with a caseload of >3000 in Hong Kong, are tested for LTBI annually until a positive result or TB diagnosis^9^. Isoniazid preventive therapy (300 mg daily for nine months) is offered to patients tested LTBI positive, after the exclusion of active TB disease. TST is the main LTBI test performed in the clinic, while a small proportion was tested by IGRA if TST was not feasible. In this study, patients diagnosed with HIV between 2002 (when LTBI testing became fully implemented) and 2013, and aged ≥18 at HIV diagnosis were selected. Their follow-up data between January 2002 and June 2017 were accessed retrospectively to allow at least three years’ follow-up. We further excluded patients with past history of TB or concurrent active TB disease (TB diagnosis within three months of HIV diagnosis).

### Data source and variables

Demographics, clinical and LTBI screening and treatment records of all HIV patients attending the clinic were accessed retrospectively. Data access was approved by Department of Health, Hong Kong Special Administrative Region Government in compliance with the Personal Data (Privacy) Ordinance. Approval of the Joint Chinese University of Hong Kong – New Territories East Cluster Clinical Research Ethics Committee (CREC) was obtained and individual consent was waived.

The collected data included demographics (date of birth, gender, ethnicity, residence), HIV diagnosis date, HIV transmission route, date and specificity of ADI, baseline body mass index (BMI), diagnosis date of diabetes mellitus (DM), HAART initiation date, date of death, TB diagnosis date, date of TB or LTBI treatment initiation with regimen, baseline and pre-HAART viral load, and longitudinal measurements of CD4, CD8, CD4/CD8 ratio, and LTBI testing results (TST or IGRA). Of note, Hong Kong has adopted the CDC 1993 classification system with modification to define AIDS. Specifically, pulmonary or cervical lymph node TB is defined as ADI only when the CD4 is below 200/µL (http://www.info.gov.hk/aids/english/surveillance/definition.htm). We have combined LTBI testing results and treatment status as an independent variable in analysis.

### Outcome variables

The main outcome of this study was TB disease development. This was obtained from records showing clinical and/or microbiological TB diagnosis with date of treatment initiation. Time to TB disease was the interval (months) from HIV diagnosis date to either TB diagnosis date or TB treatment initiation date, whichever earlier. The data end point for patients without TB was either date of death or the latest in-care date (CD4 measurement date or LTBI testing date), whichever later. Positive LTBI was defined as either an induration of ≥5 mm within 48 to 72 hours with TST or positive IGRA result^9^.

### Statistical analysis

We calculated the crude incidence rates of TB (events/100 PY) and 95% confidence interval (C.I.) assuming Poisson distribution. PY was the sum of follow-up years with either CD4 measurement or LTBI testing records. We identified factors with significantly (p < 0.05) higher risk of developing TB disease in Cox proportional hazards regression analyses. Time dependent variables of DM and ADI diagnoses were manipulated and included for analyses. In light of the significant association of HAART coverage with TB incidence^14^, we included HAART status (time dependent) and LTBI treatment status as confounders in multivariable Cox regression models. Separately, characteristics of patients ever tested and never tested for LTBI were compared, and the associated factors of the positive LTBI testing results were explored in logistic regression models. Complete-case analyses were performed. All analyses were performed in SPSS 21.

## Results

**Figure 1:**
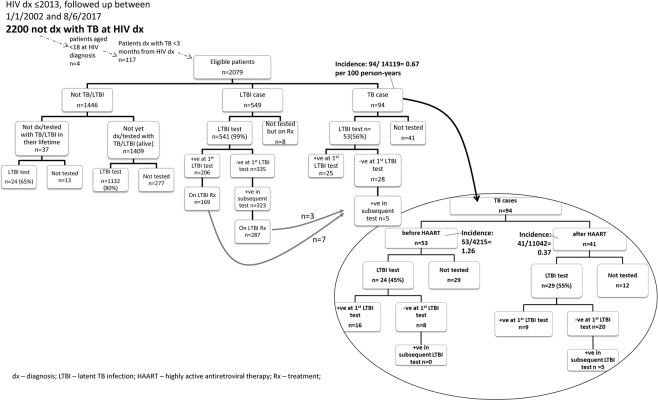
Flow chart of LTBI testing and treatment among HIV patients.

In the analysis, 2079 out of 2200 HIV patients with 14119 PY of follow-up were included (Fig. 1). The median follow-up was four years (interquartile range (IQR) = 2–8 years). A majority of patients were male (84%), local residents (87%), Chinese (74%), aged below 50 years old (90%), had initiated HAART (84%), and not been diagnosed with DM (96%) or any ADI (86%) (Table 1).

**Table 1:** Characteristics of the study population (n = 2079), stratified by development of TB disease.

	no TB (n = 1985)		TB (n = 94)		Total (n = 2079)
	n	%	n	%	N
**Socio-demographics**					
Gender					
Female	311	16%	16	17%	327
Male	1674	84%	78	83%	1752
Local residence					
No	234	12%	30	32%	264
Yes	1750	88%	64	68%	1814
Ethnicity					
Non-Chinese	503	25%	43	46%	546
Chinese	1481	75%	51	54%	1532
**Baseline conditions**					
Median HIV diagnosis year (IQR)	2009	(2006 to 2012)	2007	(2005 to 2008)	2079
Median age at HIV diagnosis (years old) (IQR)	35	(28 to 42)	34	(27 to 45)	2079
Median CD4 (/μL) (IQR)	312	(173.25 to 474)	177	(35 to 368.5)	2042
Median CD4/CD8 ratio (IQR)	0.33	(0.18 to 0.5)	0.21	(0.07 to 0.37)	2031
Median log_10_ viral load (/mL) (IQR)	4.73	(4.08 to 5.23)	5.11	(4.65 to 5.57)	2040
Diabetes mellitus diagnosis^§^					
No	1903	96%	91	97%	1994
Yes	82	4%	3	3%	85
Body mass index^§^^					
underweight	180	10%	14	20%	194
normal	929	54%	41	59%	970
marginal	316	18%	5	7%	321
overweight	253	15%	7	10%	260
obese	48	3%	2	3%	50
First ADI diagnosis^§^					
No	1705	86%	76	81%	1781
Yes	280	14%	18	19%	298
**LTBI testing**					
Ever tested LTBI					
No	298	15%	41	44%	339
Yes	1687	85%	53	56%	1740
Ever tested LTBI +ve					
No	1168	69%	23	43%	1191
Yes	519	31%	30	57%	549
First LTBI test +ve					
No	1488	88%	28	53%	1516
Yes	199	12%	25	47%	224
Subsequent LTBI test +ve					
No	1168	78%	23	82%	1191
Yes	320	22%	5	18%	325
Received LTBI treatment^&^					
No	1531	77%	84	89%	1615
Yes	454	23%	10	11%	464
LTBI test result and LTBI treatment					
LTBI test −ve and without LTBI treatment	1156	59%	23	25%	1179
LTBI test +ve but without LTBI treatment	85	4%	20	22%	105
LTBI test +ve and with LTBI treatment	434	22%	10	11%	444
Never tested and without LTBI treatment	290	15%	40	43%	330
**Conditions at HAART initiation**					
HAART^§^					
No	284	14%	53	56%	337
Yes	1701	86%	41	44%	1742
Median age (years old) (IQR)^#^	37	(31 to 45)	38	(31 to 46)	1780
Median CD4 (/μL) (IQR)^#^	233	(118 to 359)	113	(25 to 218)	1703
Median CD4/CD8 ratio (IQR)^#^	0.25	(0.14 to 0.39)	0.15	(0.04 to 0.27)	1695
Median log_10_ viral load (/mL) (IQR)^#^	4.72	(3.79 to 5.28)	5.04	(4.1 to 5.65)	1698
Deceased					
No	1943	98%	91	97%	2034
Yes	42	2%	3	3%	45

ADI – AIDS defining illness; HAART – highly active antiretroviral therapy; LTBI – latent TB infection; IQR – interquartile range.

^§^Occurrence of the respective factor after TB disease development excluded.

^&^LTBI treatment refers to the initiation of isoniazid regimen (300 mg daily) only, regardless of the exact duration of treatment as a proportion of patients had received LTBI treatment in a separate clinical service (e.g. TB and Chest Clinic) the data of which were not merged with the research dataset.

^^^BMI categories – underweight (<18.5); normal (18.5–22.9); marginal (23–24.9); overweight (25–29.9); obese (≥30). Reference: http://www21.ha.org.hk/smartpatient/MiniSites/en-US/bmi/BMI-Normal/.

### LTBI screening and identification

Eighty-four percent (1740/2079) of patients have been tested for LTBI at least once, with a median of 3 tests received while in care (IQR = 1–5). Compared to never-tested patients, a higher proportion of those tested at least once were male (85% for ever tested vs 78% for never-tested), Chinese (78% vs 51%), local residents (92% vs 62%), have been diagnosed with DM (5% vs 1%), initiated HAART (91% vs 46%), and diagnosed with ADI (15% vs 10%) (Table 2). TST was the main LTBI testing method, accounting for 95% (5411/5686) of the testing episodes.

**Table 2:** Comparison between patients never tested (n = 339) and ever tested for LTBI (n = 1740).

	never tester	%	tester	%	OR/	95% C.I./
	n		n		U test	p value
**Socio-demographics**						
Gender						
Female	73	22%	254	15%	*ref*	
Male	266	78%	1486	85%	**1.61**	**1.2 to 2.15**
Ethnicity^a^						
Non-Chinese	167	49%	379	22%	*ref*	
Chinese	171	51%	1361	78%	**3.51**	**2.75 to 4.47**
Local residence^a^						
No	128	38%	136	8%	*ref*	
Yes	210	62%	1604	92%	**7.19**	**5.43 to 9.52**
**At HIV diagnosis**						
Median age (years old); IQR	30.81	25.79 to 38.11	35.26	28.75 to 42.43	**U test**	**<0.001**
Median HIV diagnosis year; IQR	2008	2006 to 2012	2008	2006 to 2011	U test	1.00
Median CD4 (/μL); IQR^b^	322	160 to 477	304	164 to 469	U test	0.30
Median CD4/CD8 ratio; IQR^c^	0.34	0.18 to 0.58	0.32	0.17 to 0.48	U test	0.13
Median years from HIV diagnosis to last follow-up; IQR^d^	19.93	4.67 to 59.17	2.77	1.4 to 10.38	**U test**	**<0.001**
**Others**						
DM^&^						
No	337	99%	1657	95%	*ref*	
Yes	2	1%	83	5%	**8.44**	**2.07 to 34.48**
On HAART^&^						
No	184	54%	153	9%	*ref*	
Yes	155	46%	1587	91%	**12.31**	**9.39 to 16.14**
ADI diagnosis^&^						
No	306	90%	1475	85%	*ref*	
Yes	33	10%	265	15%	**1.67**	**1.14 to 2.44**
Eventually developed TB						
No	298	88%	1687	97%	*ref*	
Yes	41	12%	53	3%	**0.23**	**0.15 to 0.35**

Bold – variables have a significant association (p < 0.05) with the outcome.

a-1 missing, b-37 missing, c-48 missing, d-148 missing.

^&^Conditions after TB development were not included.

ADI – AIDS defining illness; DM – diabetes mellitus; HAART – highly active antiretroviral therapy; LTBI – latent TB infection; U test –Mann–Whitney U test.

Among HIV patients who had been screened for LTBI, 32% (549/1740) were tested positive (Table 1). The proportion of patients receiving their first LTBI test was 62% (1298/2079) before and 25% (442/1780) after HAART initiation. Around 40% of the LTBI positive cases (224/549) were identified at the first test. Among cases screened LTBI positive in subsequent tests (n = 325), the median interval between the first negative and subsequent positive tests was 42.93 months (IQR = 23.45–69.32). Among 1468 patients ever tested TST negative, 28 (2%) developed active TB.

At the first LTBI test, non-Chinese, non-local residents, previous testing in earlier calendar year, a higher CD4 level, not on HAART, longer interval before HAART initiation, and no diagnosis with any ADI were associated with a positive result (Table 3). Patients tested positive at the first LTBI test were more likely to develop TB disease, compared to those tested negative (OR = 6.68, 95% C.I. = 3.82 to 11.68), irrespective of the treatment history after screening. However, the association was no longer significant in the subsequent LTBI tests. Chinese, local residents, receiving HAART, and shorter interval on HAART were significant factors associated with positive result in the subsequent LTBI tests (Table 3).

**Table 3:** Number and proportion of patients tested positive at (a) the first and (b) subsequent LTBI tests, and the associations of factors in simple logistic regression model, unless otherwise specified.

	the first LTBI test (n = 1740)					subsequent LTBI tests^&^ (n = 1516)				
	no. of +ve	N	% of +ve	OR	95% C.I.	no. of +ve	N	% of +ve	OR	95% C.I.
**LBTI test**	224	1740	13%			325	1516	21%		
**Socio-demographics**										
Gender										
Female	30	254	12%	*ref*		43	224	19%	*ref*	
Male	194	1486	13%	1.12	0.74 to 1.69	282	1292	22%	1.18	0.82 to 1.68
Ethnicity										
Non-Chinese	71	379	19%	*ref*		48	308	16%	*ref*	
Chinese	153	1361	11%	**0.55**	**0.4 to 0.75**	277	1208	23%	**1.61**	**1.15 to 2.25**
Local residence										
No	39	136	29%	*ref*		12	97	12%	*ref*	
Yes	185	1604	12%	**0.32**	**0.22 to 0.48**	313	1419	22%	**2.00**	**1.08 to 3.72**
**Conditions at LTBI test**										
Median age (years old); (IQR)										
LTBI −ve	36	1516	(30 to 43)	*ref*	U test	40	1189	(33 to 49)	*ref*	U test
LTBI +ve	36	224	(30 to 43)	p = 0.93		42	325	(36 to 48)	p = 0.15	
Median CD4 (/μL); (IQR)										
LTBI −ve	310	1513	(175 to 470)	*ref*	U test	484	1184	(357 to 647)	*ref*	U test
LTBI +ve	378	223	(262 to 597)	**p** < **0.001**		506	325	(381 to 636)	p = 0.22	
Median CD4/CD8 ratio; (IQR)										
LTBI −ve	0.40	1512	(0.25 to 0.66)	*ref*	U test	0.56	1184	(0.40 to 0.82)	*ref*	U test
LTBI +ve	0.45	223	(0.29 to 0.63)	p = 0.16		0.58	325	(0.40 to 0.81)	p = 0.67	
**Diabetes mellitus diagnosis** ^**#**^										
No	213	1657	13%	*ref*		307	1444	21%	*ref*	
Yes	11	83	13%	1.04	0.54 to 1.99	18	72	25%	1.23	0.71 to 2.14
**on HAART** ^#^										
No	44	153	29%	*ref*		10	109	9%	*ref*	
Yes	180	1587	11%	**0.32**	**0.22 to 0.46**	315	1407	22%	**2.86**	**1.47 to 5.54**
Median months from HAART; (IQR)										
LTBI −ve	−1.43	1412	(−15.53 to 0.43)	*ref*	U test	43.60	1095	(18.17 to 71.13)	*ref*	U test
LBTI +ve	−12.08	190	(−36.79 to 0.58)	**p** < **0.001**		33.97	315	(16.73 to 59.07)	**p** = **0.001**	
**ADI diagnosis** ^**#**^										
No	205	1475	14%	*ref*		281	1270	22%	*ref*	
Yes	19	265	7%	**0.48**	**0.29 to 0.78**	44	246	18%	0.77	0.54 to 1.09
**Eventually developed TB**										
No	199	1687	12%	*ref*		320	1488	22%	*ref*	
Yes	25	53	47%	**6.68**	**3.82 to 11.68**	5	28	18%	0.79	0.3 to 2.1

Bold – variables have a significant association (p < 0.05) with the outcome.

^#^Occurrence of the respective factor after TB disease development excluded.

^&^For patients with all negative LTBI testing results, the last test was selected for analysis.

ADI – AIDS defining illness; HAART – highly active antiretroviral therapy; LTBI – latent TB infection; U test –Mann–Whitney U test.

During the study period, 549 patients were identified as having LTBI, of which 85% had been put on LTBI treatment (Fig. 1). Two percent (10/464) of patients who received LTBI treatment eventually developed TB disease, after a median interval (between LTBI treatment start date to TB disease development date) of 24.9 months (IQR = 13.19–55.4).

### TB incidence

With 14119 PY of follow-up, the TB incidence was 0.67 /100 PY (94/14119, 95% C.I. = 0.54 to 0.81/100 PY) (Fig. 1). Among 94 TB cases, only 56% (53/94) had been tested for LTBI (52 by TST and 1 by IGRA), and 30 of which were tested positive (Table 1, Fig. 1). Thirty percent (28/94) had history of negative baseline LTBI testing results before TB disease, including five who subsequently tested positive, with a median interval of 16.63 months (IQR = 4.93–37.31).

Around half (53/94) of the TB cases were diagnosed before HAART initiation, giving a TB incidence of 1.26 /100 PY (53/4215, 95% C.I. = 0.95 to 1.63/100 PY) (Fig. 1). Only half (24/53) of the pre-HAART TB cases had history of LTBI test before TB diagnosis, and 67% (16/24) of them were tested LTBI positive at the first test. Eight cases who tested LTBI negative were eventually diagnosed with TB. After HAART initiation, TB incidence dropped by 70% (41/11042 = 0.37 /100 PY, 95% C.I. = 0.27 to 0.50/100 PY). Some half (29/41) of them had been screened for LTBI. Only nine patients were tested LTBI positive at the first test and five more tested positive in subsequent tests.

Adjusted by HAART status, the adjusted hazard ratio (aHR) of TB disease development among patients tested positive at the first LTBI test (aHR = 6.18, 95% C.I. = 3.57 to 10.73) was much higher than those ever tested LTBI positive (aHR = 2.54, 95% C.I. = 1.47 to 4.37) (Table 4). However, patients screened positive in subsequent LTBI tests were not at significantly higher risk of TB disease development than those screened negative. LTBI treatment was a significant variable of TB disease development (aHR = 0.34, 95% C.I. = 0.18 to 0.65). Compared to patients tested LTBI negative, patients without LTBI treatment (either LTBI positive or never tester) were at higher risk of TB disease. Patients tested LTBI positive followed by LTBI treatment were not at significantly higher risk than patients tested LTBI negative. However, LTBI treatment was not a strong confounder as the HR of other variables remained similar after adjustment, except ADI diagnosis (Table 4).

**Table 4:** Factors associated with TB disease development in bivariable and multivariable cox regression models.

	Bivariable model		adjusted by HAART status (time dependent)		adjusted by LTBI treatment	
	HR	95% C.I.	aHR	95% C.I.	aHR	95% C.I.
**Socio-demographics**						
Male gender	0.88	0.51 to 1.5	0.88	0.51 to 1.5	0.90	0.53 to 1.55
Local residence	**0.20**	**0.13 to 0.31**	**0.23**	**0.15 to 0.37**	**0.21**	**0.14 to 0.33**
Chinese	**0.34**	**0.23 to 0.51**	**0.38**	**0.25 to 0.57**	**0.37**	**0.24 to 0.55**
**Baseline conditions**						
HIV diagnosis year	**0.90**	**0.84 to 0.96**	**0.92**	**0.86 to 0.98**	**0.89**	**0.83 to 0.95**
Age at HIV diagnosis (years old)	0.998	0.98 to 1.02	1.01	0.99 to 1.03	1.001	0.98 to 1.02
First CD4 (/μL)	**0.998**	**0.997 to 0.999**	**0.996**	**0.995 to 0.998**	**0.998**	**0.997 to 0.999**
First CD4/CD8 ratio	**0.09**	**0.03 to 0.27**	**0.03**	**0.01 to 0.11**	**0.10**	**0.03 to 0.29**
First log_10_ viral load (/mL)	**1.58**	**1.24 to 2.02**	**1.93**	**1.48 to 2.51**	**1.58**	**1.25 to 2.01**
Diabetes mellitus diagnosis^§^	1.05	0.33 to 3.32	1.25	0.39 to 3.97	1.54	0.37 to 6.43
Body mass index^						
underweight	*ref*		*ref*		*ref*	
normal	0.56	0.3 to 1.02	**0.53**	**0.29 to 0.97**	0.56	0.3 to 1.02
marginal	**0.21**	**0.08 to 0.58**	**0.20**	**0.07 to 0.55**	**0.22**	**0.08 to 0.6**
overweight	**0.35**	**0.14 to 0.87**	**0.33**	**0.13 to 0.82**	**0.37**	**0.15 to 0.91**
obese	0.53	0.12 to 2.34	0.48	0.11 to 2.13	0.59	0.13 to 2.62
First ADI diagnosis^§^	**5.59**	**3.71 to 8.41**	**12.46**	**7.66 to 20.24**	**11.11**	**5.91 to 20.88**
**LTBI testing**						
Ever tested LTBI	**0.14**	**0.09 to 0.21**	**0.15**	**0.1 to 0.23**	/	
Ever tested LTBI +ve	**2.60**	**1.51 to 4.48**	**2.54**	**1.47 to 4.37**	/	
First LTBI test +ve	**6.47**	**3.77 to 11.10**	**6.18**	**3.57 to 10.73**	/	
Subsequent LTBI test +ve	0.68	0.26 to 1.79	0.68	0.26 to 1.78	/	
Received LTBI treatment^&^	**0.33**	**0.17 to 0.64**	**0.34**	**0.18 to 0.65**	/	
LTBI test result and LTBI treatment						
LTBI test -ve and without LTBI treatment	*ref*		*ref*			
LTBI test +ve but without LTBI treatment	**10.71**	**5.88 to 19.5**	**10.50**	**5.75 to 19.17**	/	
LTBI test +ve and with LTBI treatment	1.01	0.48 to 2.12	1.01	0.48 to 2.11	/	
never tested and without LTBI treatment	**11.47**	**6.85 to 19.19**	**10.87**	**6.34 to 18.61**	/	
**Conditions at HAART initiation**						
HAART^§^	**0.45**	**0.29 to 0.71**	/		**0.46**	**0.3 to 0.73**
Age (years old)	1.02	0.99 to 1.05	/		1.02	0.99 to 1.05
CD4 (/μL)	**0.995**	**0.99 to 0.998**	/		**0.99**	**0.99 to 0.997**
CD4/CD8 ratio	**0.01**	**0.001 to 0.13**	/		**0.007**	**0.001 to 0.08**
log10 viral load (/mL)	1.10	0.84 to 1.43	/		1.09	0.83 to 1.43

Bold – variables have a significant association (p < 0.05) with the outcome.

ADI – AIDS defining illness; HAART – highly active antiretroviral therapy; LTBI – latent TB infection; IQR – interquartile range.

HR – hazard ratio estimated in bivariable cox regression model: model event = TB disease, time variable = from HIV diagnosis to the end point (either TB date or latest in care date (largest date of CD4 collection, PPD collection, death, LTBI date, TB date, HAART initiation date), whichever earlier).

aHR – adjusted hazard ratio estimated in multivariable cox regression model with the confounder.

^BMI categories – underweight (<18.5); normal (18.5–22.9); marginal (23–24.9); overweight (25–29.9); obese (≥30). Reference: http://www21.ha.org.hk/smartpatient/MiniSites/en-US/bmi/BMI-Normal/.

^&^LTBI treatment refers to the initiation of isoniazid regimen (300 mg daily) only, regardless of the exact duration of treatment as a proportion of patients had received LTBI treatment in a separate clinical service (e.g. TB and Chest Clinic) the data of which were not merged with the research dataset.

^§^Occurrence of the respective factor after TB disease development excluded.

## Discussion

This is a longitudinal study conducted in a HIV clinic with fairly satisfactory compliance to established LTBI screening and treatment guidance, in the setting of an intermediate TB burden city. The compliance figures of LTBI screening in Hong Kong (84%) appeared to be higher than that of other developed countries (<70%)^11–13,15^. Nonetheless, the prevalence of LTBI in this study (32%) was similar to nearby Asian places (Iran, Korea, Thailand, Taiwan)^21,22^, and the proportion of TB cases with LTBI testing history (56%) was close to that of a Canadian study (61%)^14^.

Our results suggested that baseline LTBI screening for all HIV patients is an important step for reducing TB incidence. Since HIV infection is a strong predictor of TB reactivation^23^, the first LTBI screening allows vulnerable patients to be promptly identified to prevent reactivation^9,24^. Generally, our results showed that HIV patients never tested for LTBI and those tested positive at the first instance were at higher risk of TB disease development, consistent with the results of a study in Spain^25^. On the other hand, non-local residents and non-Chinese were shown to be at higher risk of TB disease development, and were more likely to be identified at the first LTBI screening. The finding echoed the results of studies that non-local residents from high TB burden places were at higher risk of LTBI and TB disease development^12,26^.

In our study, there was a drop of TB incidence (in HIV care) by three-folds following HAART initiation. Patients with higher CD4 and/or CD4/CD8 ratio were at lower risk of TB disease development, as observed in our and previous studies^24,27,28^, as a result of immune recovery^18,19^. The results were consistent with those reported in a study showing the four times higher TB incidence among heterosexual HIV patients not on HAART^29^, and conclusion from a systematic review^19^. Of note, the TB incidence among patients after HAART initiation was 0.37/100 PY, much lower than that of another local study (0.59/100 PY) on patients with antiretroviral therapy between 1989 and 2011 in the same clinic^9^. This was partly due to the continuous clinical and programmatic improvement of HIV treatment in the recent years^30,31^. Locally, HAART was implemented in Hong Kong since 1996/97, and HAART initiation threshold raised from 200/μL to ≤350/μL in 2010, changing to a CD4-unguided approach in recent years^32,33^. We, however, acknowledge that despite its potency, HAART could not completely restore the body’s responses to TB-antigens nor eliminate the risk of TB progression^34^.

The improvement of immune status after HAART initiation could reduce the probability of false negative LTBI testing results. The positive rate of LTBI at very low CD4 level (<100/μL) was particularly low, as shown also in a multicentre European cohort study and other studies^21,35^. We observed a rise of positive LTBI rate in association with the improving immune status (CD4 and CD4/CD8 ratio) among patients in both the first and subsequent LTBI tests. When patients on HAART with satisfactory immune recovery gave LTBI negative results, subsequent annual testing would become less cost-effective. The main rationales for repeated LBTI screening are, arguably: 1) diagnosis of new TB infection acquired after previous LTBI screening(s); and/or 2) identifying LTBI patients who had given false negative result in the past due to the underlying immunodeficiency. From our 15 years’ data, only five TB cases was identified in subsequent LTBI screenings. Non-local residents or non-Chinese were also not at higher risk of positive LTBI on subsequent testing. Our observations provided evidence that the probability of new infection after the first LTBI screening was very low among HIV patients on HAART.

Our study carried some limitations. First, the study population was relatively young (98% aged below 65). Therefore, the contribution of TB reactivation by age progression might not be obvious. There’s a chance that some incident cases had resulted from newly acquired infections. Second, false-positive LTBI testing could occur due to intrinsic intra-individual variability of test result as a result of past Bacillus Calmette–Guérin (BCG) vaccination which is routinely administered on all local newborns. On the other hand, boosting effect (increased tuberculin reactions without new infection) with repeat TST could be a confounder despite the long interval between tests of at least 1 year^36^. Third, the predictive power of LTBI screening and the effect of LTBI treatment on TB reactivation could not be directly estimated under the current study design. Lastly, our study was conducted in an intermediate TB burden city. Extrapolation of the results to high burden developing countries would require further studies for validation. In addition, 95% of LTBI test results were based on TST instead of IGRA. The association between CD4 and test results might not be applicable when IGRA is the main testing method. Likewise, the context of drug-resistance had not been investigated as the study dataset did not include relevant information for the analyses.

In conclusion, our findings highlighted the importance of baseline LTBI screening for all HIV patients for minimising occurrence of clinical TB diseases and for reducing TB incidence in the population. However, with early HAART initiation among HIV patients, re-evaluation of the strategy of annual LTBI screening is needed. The risk factors identified in this study might provide useful references for the re-evaluation.

## Data Availability

The datasets analysed during the current study are not publicly available because the data are owned by third parties. Access to these data and permission could be inquired through Department of Health, Hong Kong Special Administrative Region Government.
